# Geographical patterns and determinants of migraine in persons aged ten years or older living in Sweden from 2015 to 2023: a nationwide cross-sectional study

**DOI:** 10.1186/s10194-026-02287-1

**Published:** 2026-02-19

**Authors:** Emily White Johansson, Ahmed Nabil Shaaban, Mattias Linde, Mathias Mattsson, Lode van der Velde, Sofie Gustafsson, Johan Holm, Christina Dalman, Emilie E. Agardh

**Affiliations:** 1 Department of Global Public Health, Karolinska Institutet, Stockholm, Sweden; 2 Global Health and Migration Unit, Department of Women’s and Children’s Health, Uppsala University, Akademiska sjukhuset, Uppsala, 751 85 Sweden; 3 Department of Neuromedicine and Movement Science, Norwegian University of Science and Technology (NTNU), Trondheim, Norway; 4 Regional Migraine Unit, Sahlgrenska University Hospital, Gothenburg, Sweden; 5 Pfizer AB, Stockholm, Sweden

**Keywords:** Migraine, Residential deprivation, Sweden

## Abstract

**Background:**

Migraine is a major cause of disability affecting a person’s health, well-being, working life and social relationships. In Sweden, it was previously estimated that approximately one in eight people experience migraine with lower socioeconomic groups disproportionately affected. Yet there is a lack of recent evidence on the burden and geographic distribution of migraine within Sweden including by small-area deprivation.

**Methods:**

A nationwide register-based cross-sectional study was conducted of persons ten years or older in Sweden on 31 December 2023 and who were diagnosed with or prescribed drugs for migraine during the study period (2015–2023). Crude and age-standardized migraine rates per 1,000 persons were tabulated nationally and sub-nationally by region and small-area deprivation measured using the Index for Multiple Deprivation in Sweden (IMDIS). Logistic regression models quantified the association between small-area deprivation level and other covariates (age, sex, area of residence, birthplace) on migraine. Among migraine patients, the healthcare source for the first recorded diagnosis was compared at national and regional levels as well as by patient characteristics.

**Results:**

A total of 372,926 people aged ten years or older had recorded migraine during the study period corresponding to a rate of 43.7 cases per 1,000 persons. Higher migraine occurrence was found in the least versus the most deprived areas (OR: 1.05, 95% CI: 1.04–1.06). Among migraine patients, the first diagnosis occurred in primary (27.2%), specialized outpatient (13.9%), or inpatient (3.8%) care while 55.2% were prescribed drugs without a recorded diagnosis in the study period. There was significant variation in the source of the first migraine diagnosis across regions and by the patient’s age, birthplace and area of residence.

**Conclusions:**

We found low migraine rates using administrative healthcare registry data compared to higher estimates reported in previous population surveys in Sweden. The higher prevalence of migraine among people in better-resourced areas compared to more deprived areas suggests potential healthcare gaps for migraine patients across socioeconomic contexts. Significant variation in the source of the first migraine diagnosis by region and patient characteristics merits further investigation. Overall findings suggest healthcare gaps for migraine patients in Sweden with uneven practices across regions and socioeconomic contexts.

**Supplementary Information:**

The online version contains supplementary material available at 10.1186/s10194-026-02287-1.

## Background

Migraine is the second leading cause of disability worldwide and it affects more than one billion annually [1]. It is considered a major public health problem given its impact on a person’s health and well-being, and the disruptions it may further cause in social relationships, ability to work, and income loss [2–4].

In Sweden, migraine has been estimated to affect at least one in eight people with women and people in lower socioeconomic groups disproportionately affected [5, 6]. However, burden estimation for migraine is a well-recognized challenge in many high-income countries given significant healthcare gaps for migraine patients [7–11]. Evidence suggests that approximately four of ten people with migraine seek health care for the condition, and among those care-seekers, only about one-quarter subsequently receive a correct diagnosis [7]. Migraine-specific drugs are also under-utilized [8–10, 12]. A similar pattern has been found in previous research from Sweden with healthcare gaps for migraine patients and unequal care across regions and by socioeconomic status [5, 6, 13, 14].

Yet there remains a lack of recent evidence on the burden and geographic distribution of migraine cases within Sweden. While individual-level risk factors for migraine (e.g., age, sex, socioeconomic status and genetic factors) are well-established [15], the role of contextual factors such as residential deprivation is less understood [15]. Residential deprivation is linked to stress levels, crime and violence, access to quality services, social cohesion, among other mechanisms that could potentially impact migraine risk [16–18]. A more granular description of deprivation for small areas within a society may better capture variations in health outcomes, including migraine, which could potentially be obscured at higher levels of geographical aggregation.

To help fill this evidence gap, this study aims to describe the burden and distribution of migraine in persons aged ten years or older living in Sweden from 2015 to 2023 using administrative registers, including by small geographic areas with different deprivation levels. As a secondary aim, we compared the healthcare level for the first migraine diagnosis by region and patient characteristics.

## Methods

We conducted a cross-sectional study linking multiple national administrative registers with regional primary care datasets to describe the geographic patterns of migraine in persons aged ten years or older living in Sweden in 2015 − 2013 and its association with small-area deprivation. We also compared the healthcare level for the first migraine diagnosis by region and patient characteristics across primary, specialist outpatient and inpatient care in 2015–2017 when data were available.

### Data sources

The study linked national and regional administrative registers through a Swedish personal identification number unique to each resident. These registers included: (1) the Total Population Register with population and household statistics for all Swedish residents; (2) the National Prescribed Drug Register containing all prescribed drugs dispensed at pharmacies in Sweden; (3) the National Patient Register providing data on the disorders and treatments managed through in-patient admission and specialized outpatient care in Sweden; and (4) the Primary Healthcare Registers providing data on the disorders and treatments managed through primary healthcare centers at the regional level. All registers had national coverage for the duration of the study except the primary healthcare registers that were only available for the years 2015 to 2017 and for 17 of 21 regions representing 89% of the Swedish population. The study received approval from the Ethical Review Authority in Sweden (2018/1339-31/5, 2018/2292-32, 2019–02185, 2021 − 00657, 2022-03111-02, 2023-07509-02, 2024-02816-02).

### Study population

We identified all persons registered in Sweden throughout the study period from 2015 to 2023 who had a personal identification number assigned at birth or immigration, and a primary address assigned to one of 5,984 small geographical divisions established by Statistics Sweden (DeSO) (*n* = 8,728,980) [19]. We excluded people that were under the age of ten years on 31 December 2023 (*n* = 200,782) since migraine onset typically occurs in adolescence [20]. In this way, the numerator includes all persons aged ten years or older who had a migraine exposure during the multiyear study period although the denominator will also include persons under ten years old who reached that age by the final study date. The final study population included 8,528,198 individuals.

### Migraine definition

Migraine was defined as the first recorded diagnosis date in primary, specialist outpatient, or inpatient care, and if no diagnosis was recorded during the study period, the first prescription date of a dispensed drug was used. Table S1 lists the diagnosis for migraine (G43) and prescription codes (triptans and calcitonin gene-related peptide (CGRP) inhibitors) that measured the exposure based on the Swedish adaptation of the International Statistical Classification of Diseases and Related Health Problems, 10th version (ICD-10-SE) and the Anatomical Therapeutic Chemical (ATC) classification system, respectively. Migraine diagnoses were derived from any inpatient admission or specialist outpatient visit throughout the study period, and any primary care visit in 17 of 21 regions from 2015 to 2017 when data were available. There were 31,300 people diagnosed with migraine in primary care that had no visit date (day and month). For these cases, we imputed 18,349 (59%) diagnosis dates using the earliest prescription date in the file year. For the remaining 12,951 (41%) people without a visit date (day and month), the midpoint date in the file year was used. For migraine treatment, if the prescription date occurred prior to 1 January 2015, we used the first date of dispensation for the drug during the study period. Study participants without recorded migraine during the study period comprised the comparison group.

### Small geographic areas

In 2018, Statistics Sweden developed new demographic statistical areas (DeSO) that divided the country into approximately 6,000 small geographic areas to help facilitate monitoring of segregation and socioeconomic conditions over time [19]. These small geographic areas were created using electoral districts and urban areas while accounting for natural borders such as roads, waterways and railroads to the extent possible. There was a median of 1,502 (IQR: 1,263-1,763) study participants living in each of the 5,984 DeSO areas in our analysis.

### Small-area deprivation

Small-area deprivation was estimated using an Index for Multiple Deprivation in Sweden (IMDIS) applied to each of the 5,984 DeSO areas in the year 2015. IMDIS has been previously described elsewhere [21]. Briefly, the index aims to capture the deprivation level of each DeSO through a composite measure including 15 indicators across the following four domains: income and capital, education, employment, and housing. Indicators were spatially smoothed to reduce small sample bias and enhance robustness. Domains were formed through weighted aggregation of indicators and combined into an IMDIS score using defined weights. DeSO areas were ranked from least to most deprived and categorized into quartiles at the 25th, 50th, and 75th percentiles (very low, low, high, and very high deprivation levels).

Each study participant with migraine was assigned to the deprivation level of the DeSO for their primary address in the Total Population Register in the year of first migraine exposure. Participants not exposed to migraine were assigned the most frequent DeSO (mode) for their primary address if they moved between DeSO areas during the study period. The deprivation level assigned to each DeSO in the year 2015 was assumed to remain unchanged throughout the study period. A recent study found broad stability in Stockholm neighborhoods between 1990 and 2015 with 80% of these neighborhoods remaining in the same socioeconomic profile during this 25-year period [22].

### Other covariates

We selected covariates for inclusion in regression models based on empirical evidence of their relationship with residential deprivation and migraine occurrence, as well as based on data availability in the dataset. Region and area of residence (urban/peri-urban/rural) as defined by Statistics Sweden was derived from the DeSO code assigned to each study participant. Other regression covariates included sex (male or female), birthplace (Sweden, Nordic outside Sweden, European Union (EU28) outside Nordic, Europe outside EU28, or another birthplace), and age at the study end date (continuous). The person’s age at the time of first migraine exposure was used to calculate age-standardized migraine rates and to analyze the source of migraine diagnosis by age group.

### Statistical analyses

We calculated crude and age-standardized migraine rates per 1,000 persons aged ten years or older on 31 December 2023 who were living in Sweden from 1 January 2015 to 31 December 2023. Age-standardized rates were generated by applying the age-specific migraine rates in sub-national areas (region, area of residence, and small-area deprivation level) to the age distribution of the Swedish population in 2023. We tabulated age-standardized migraine rates for each DeSO, which were empirically categorized into quartile groupings. The categorized migraine rates for each DeSO were mapped alongside its assigned small-area deprivation level.

To examine the association between small-area deprivation and migraine, we initially evaluated the extent of variation in migraine cases across small geographic areas using a random-intercept model with a DeSO identifier as the random intercept. A small proportion of variance in migraine cases was attributed to differences between DeSO areas (intra-class correlation (ICC) = 0.005) suggesting that a standard logistic regression model was suitable to estimate odds ratios (OR) with 95% confidence intervals (CI) for associations between migraine and other covariates in our study. We estimated crude ORs for the association between small-area deprivation and migraine and subsequently adjusted the model for all covariates previously described. We used a Wald test to determine if small-area deprivation level modified the relationship between other covariates and the migraine outcome, and the final model was stratified by small-area deprivation to further explore these results. A subset analysis was conducted among people with their first recorded migraine diagnosis in the years when primary care data were available (2015–2017) to examine the source of the first migraine diagnoses. The level of statistical significance was set to 0.05. We analyzed data using Stata 18.1 (Stata Corp., College Station, TX).

### Sensitivity analyses

We conducted two sensitivity analyses for this study. In the first sensitivity analysis, the definition of migraine was restricted to diagnoses only and prescribed migraine drugs (without a diagnosis in the study period) were omitted from the definition. In the second sensitivity analysis, we examined associations between migraine and small-area deprivation level without primary healthcare data since these data were not available throughout the study period and limited to specific years (2015 to 2017) and regions (17 of 21).

## Results

### Migraine rates

**Fig. 1:**
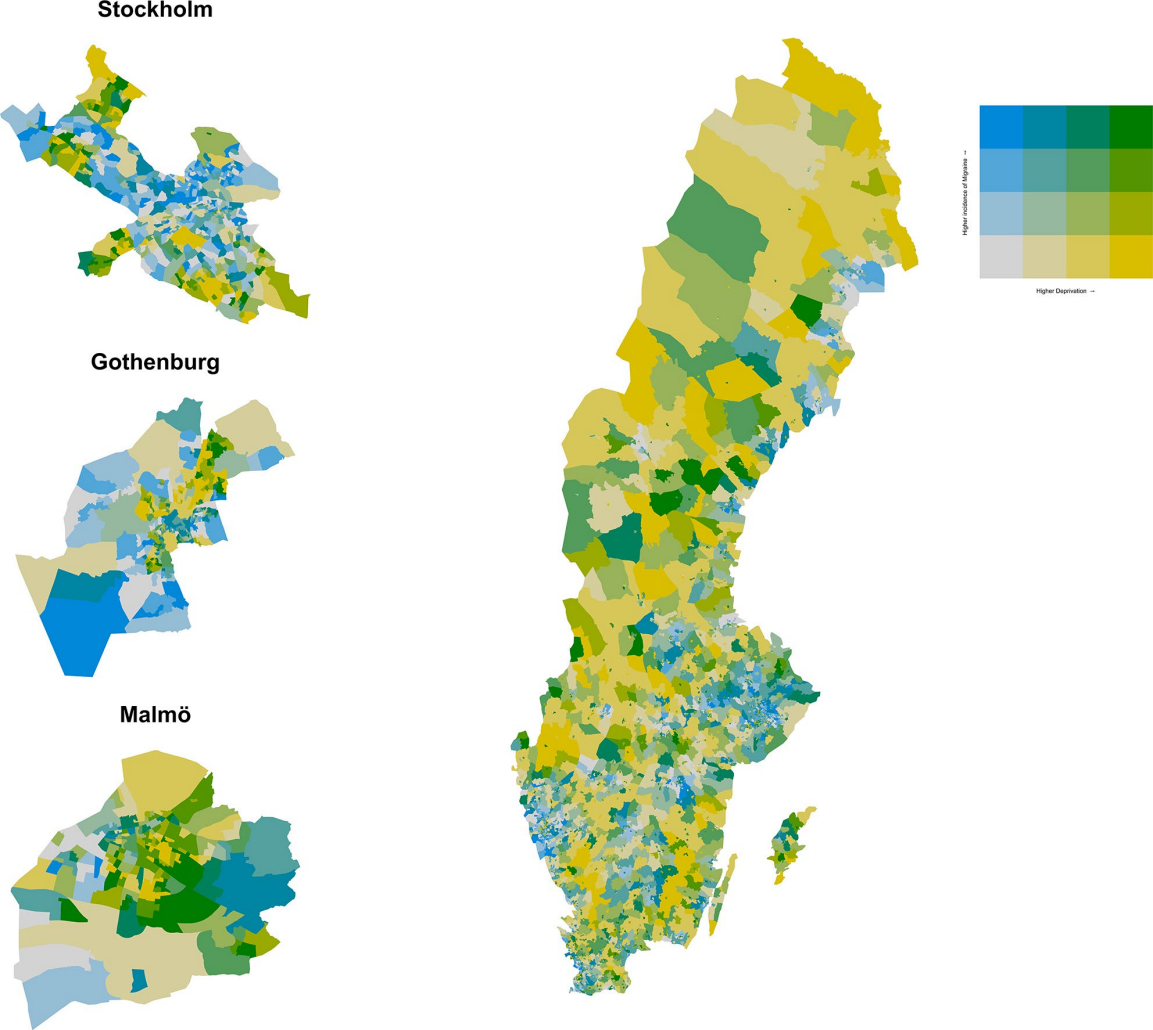
Migraine rates per 1,000 persons aged ten years or older in Sweden in 2015–2023, by small-area deprivation ^1^. ^1^ Age-standardized migraine rates were tabulated for each DeSO and empirically categorized into quartiles groupings. Small-area deprivation levels were tabulated for each DeSO using the Index for Multiple Deprivations in Sweden (IMDIS), which were categorized into quartiles at the 25th, 50th, and 75th percentiles (very low, low, high, very high deprivation)

The crude migraine rate was 43.7 cases per 1,000 persons aged ten years or older living in Sweden from 2015 to 2023 (Table 1). Across regions, age-standardized migraine rates varied from 40.4 cases per 1,000 persons aged ten years or older in Norrbotten to 49.1 in Gotland. Age-standardized migraine rates were 43.9 in urban areas, 44.8 in peri-urban areas and 43.2 in rural areas. The age-standardized migraine rate in areas with very high deprivation levels was 42.9 compared to 43.7 in very low deprivation areas (Fig. 1).

**Table 1:** Migraine rates per 1,000 persons aged ten years or older in Sweden in 2015–2023

	Age-standardized rate	Crude rate	Migraine cases	Persons aged 10 years or older on 31 December 2023
	per 1,000	per 1,000	*N*	*N*
**National**	**43.7**	**43.7**	**372 926**	**8 528 198**
**Small-area deprivation**				
Very low deprivation	43.7	43.9	98 849	2 251 388
Low deprivation	44.4	44.2	96 916	2 192 330
High deprivation	44.1	43.1	91 080	2 113 810
Very high deprivation	42.9	43.7	86 081	1 970 670
**Area of residence**				
Urban	43.9	44.7	287 216	6 427 223
Peri-urban	44.8	42.9	31 675	737 910
Rural	43.2	39.6	54 035	1 363 065
**Region of residence**				
Stockholm	45.2	46.8	91 073	1 945 231
Uppsala	46.3	46.9	14 659	312 429
Södermanland ^1^	42.1	40.6	9 900	244 114
Östergötland	41.8	41.8	16 218	388 142
Jönkoping	43.8	43.3	13 012	300 653
Kronoberg ^1^	46.1	45.4	7 410	163 226
Kalmar	44.5	41.8	8 502	203 473
Gotland	49.1	45.8	2 316	50 608
Blekinge	42.7	41.0	5 432	132 535
Skåne	43.6	43.8	48 976	1 117 910
Halland ^1^	43.1	42.0	11 703	278 930
Västra götaland	43.5	43.8	62 750	1 431 275
Värmland	40.6	38.7	9 114	235 738
Örebro	40.8	40.4	10 190	252 410
Västmanland	46.4	45.4	10 376	228 450
Dalarna	44.5	42.1	10 189	242 109
Gävleborg	42.4	40.3	9 754	241 845
Västernorrland	46.7	44.6	9 287	208 111
Jämtland	42.1	40.5	4 446	109 875
Västerbotten ^1^	41.5	41.3	9 431	228 341
Norrbotten	40.4	38.5	8 188	212 793

^1^ Primary healthcare data was not available for these regions. All other regions had primary healthcare data available for the years 2015 to 2017 only

### Associations with migraine occurrence

Among study participants, migraine occurrence was associated with female gender, urban residence, and birthplace outside the European Union (EU28) versus native-born Swedes (Table 2). In adjusted regression models, compared to areas with very high deprivation levels, there was higher migraine prevalence associated with residing in very low (OR: 1.05, 95% CI: 1.04–1.06), low (OR: 1.07, 95% CI: 1.06–1.08) and high deprivation areas (OR: 1.04, 95% CI: 1.03–1.06) (Table 3).

**Table 2:** Characteristics of study participants with or without migraine

	Study participants			
	Migraine		No Migraine	
	*N*	%	*N*	%
**Total**	**372 926**	**4.4**	**8 155 272**	**95.6**
**Small-area deprivation level**				
Very low deprivation	98 849	4.4	2 152 539	95.6
Low deprivation	96 916	4.4	2 095 414	95.6
High deprivation	91 080	4.3	2 022 730	95.7
Very high deprivation	86 081	4.4	1 884 589	95.6
**Area of residence**				
Urban	287 216	4.5	6 140 007	95.5
Peri-urban	31 675	4.3	706 235	95.7
Rural	54 035	4.0	1 309 030	96.0
**Age**,** median (IQR)**	46	(33–58)	48	(30–66)
**Sex**				
Male	93 017	2.2	4 157 734	97.8
Female	279 909	6.5	3 997 538	93.5
**Birthplace**				
Sweden	307 234	4.3	6 877 465	95.7
Nordic not Sweden	6 913	3.8	174 711	96.2
EU28 not Nordic	10 365	4.2	239 351	95.8
Europe not EU28	10 810	5.2	197 244	94.8
Other birthplace	37 604	5.3	666 501	94.7

**Table 3:** Association between small-area deprivation and other covariates on migraine occurrence among study participants

	Study participants							
	Crude				Adjusted			
	OR	95% CI		*p*-value	OR	95% CI		*p*-value
**Small-area deprivation**								
Very low deprivation	1.01	1.00	1.01	0.260	1.05	1.04	1.06	< 0.001
Low deprivation	1.01	1.00	1.02	0.009	1.07	1.06	1.08	< 0.001
High deprivation	0.99	0.98	1.00	0.003	1.04	1.03	1.06	< 0.001
Very high deprivation	1.00				1.00			

### Stratification of migraine risk by small-area deprivation

We found significant interactions between small-area deprivation level with other covariates (Table S2). Specifically, migraine prevalence associated with urban versus rural residence in the most deprived areas was 1.12 (95% CI: 1.09–1.16) while it was non-significant in least deprived areas (OR: 1.02, 95% CI: 0.99–1.05) (Table 4). Women had increasingly stronger migraine prevalence compared to men in increasingly deprived areas (very low deprivation: 3.00, 95% CI: 2.96–3.05 versus very high deprivation: 3.30, 95% CI: 3.24–3.35).

**Table 4:** Association between risk factors and migraine among study participants stratified by small-area deprivation

	Very low deprivation				Low deprivation				High deprivation				Very high deprivation			
	Adjusted				Adjusted				Adjusted				Adjusted			
	OR	95% CI		p-value	OR	95% CI		p-value	OR	95% CI		p-value	OR	95% CI		p-value
**Area of residence**																
Urban	1.02	0.99	1.05	0.163	1.03	1.02	1.05	< 0.001	1.11	1.09	1.13	< 0.001	1.12	1.09	1.16	< 0.001
Peri-urban	1.00	0.96	1.03	0.896	1.01	0.98	1.04	0.457	1.10	1.07	1.13	< 0.001	1.15	1.11	1.19	< 0.001
Rural	1.00				1.00				1.00				1.00			
**Age (continuous)**	1.00	1.00	1.00	0.001	1.00	1.00	1.00	< 0.001	0.99	0.99	0.99	< 0.001	0.99	0.99	1.00	< 0.001
**Sex**																
Male	1.00				1.00				1.00				1.00			
Female	3.00	2.96	3.05	< 0.001	3.13	3.09	3.18	< 0.001	3.21	3.16	3.26	< 0.001	3.30	3.24	3.35	< 0.001
**Birthplace**																
Sweden	1.00				1.00				1.00				1.00			
Nordic not Sweden	0.91	0.86	0.95	< 0.001	0.86	0.82	0.91	< 0.001	0.87	0.83	0.92	< 0.001	0.88	0.84	0.93	< 0.001
EU28 not Nordic	1.02	0.99	1.07	0.188	0.95	0.91	0.99	0.018	0.97	0.93	1.01	0.118	0.99	0.96	1.03	0.612
Europe not EU28	1.24	1.17	1.31	< 0.001	1.25	1.19	1.32	< 0.001	1.25	1.20	1.31	< 0.001	1.24	1.21	1.28	< 0.001
Other birthplace	1.21	1.18	1.25	< 0.001	1.23	1.20	1.26	< 0.001	1.24	1.21	1.27	< 0.001	1.29	1.26	1.31	< 0.001

### Sensitivity analysis

In the first sensitivity analyses with migraine defined only based on diagnosis codes, the migraine rate was 18.2 cases per 1,000 persons with age-standardized migraine rates varying across regions from 12.2 cases per 1,000 in Örebro to 23.8 and 23.2 in Stockholm and Gotland, respectively (Table S3). Age-standardized migraine rates were highest in urban areas (18.6) compared to peri-urban (17.2) and rural areas (16.8), and similarly higher in least deprived areas (19.5) compared to low (18.8), high (17.5) and very high deprivation levels (17.0). There were no significant differences in associations between covariates with the diagnosed migraine outcome in the sensitivity analysis versus results from the main analysis (Tables S4 and S5). In the second sensitivity analysis that excluded primary healthcare data, there was no significant difference in migraine prevalence associated with living in the least versus most deprived areas (OR: 1.00, 95% CI: 0.99–1.01) and this result differed from the main analysis (Table S6).

### Source of first migraine diagnosis

We conducted a subset analysis of study participants whose first recorded migraine was between 2015 and 2017 to describe patterns in the source of diagnosis. Among the 189,150 migraine patients, 27.2% were first diagnosed in primary care, 13.9% in specialist outpatient care, 3.8% during inpatient admission, and 55.2% received a prescribed drug without a diagnosis recorded during the study period (Table 5). In these cases, the diagnosis may have occurred at an earlier time with migraine treatment continuing into the study period such that it is captured in the National Prescription Register without a concurrent diagnosis.

**Table 5:** Source of the first migraine diagnosis among study participants in 2015–2017 ^1^

	Total	Source of diagnosis						Prescription only (no diagnosis)	
		Primary		Specialist outpatient		Inpatient			
	N	N	%	N	%	N	%	N	%
**Total**	**189 150**	**51 408**	**27.2**	**26 240**	**13.9**	**7 184**	**3.8**	**104 318**	**55.2**
**Small-area deprivation**									
Very low deprivation	50 366	16 023	31.8	6 142	12.2	1 736	3.4	26 465	52.5
Low deprivation	49 254	13 349	27.1	6 876	14.0	1 891	3.8	27 138	55.1
High deprivation	45 900	10 514	22.9	6 926	15.1	1 856	4.0	26 604	58.0
Very high deprivation	43 630	11 522	26.4	6 296	14.4	1 701	3.9	24 111	55.3
**Age**									
10–19 years	18 122	6 880	38.0	5 670	31.3	332	1.8	5 240	28.9
20–29 years	28 469	9 943	34.9	4 365	15.3	943	3.3	13 218	46.4
30–39 years	34 657	10 558	30.5	4 740	13.7	1 272	3.7	18 087	52.2
40–49 years	45 167	11 456	25.4	4 975	11.0	1 525	3.4	27 211	60.2
50–59 years	35 637	7 611	21.4	3 303	9.3	1 285	3.6	23 438	65.8
60 years or older	27 098	4 960	18.3	3 187	11.8	1 827	6.7	17 124	63.2
**Sex**									
Male	45 625	11 797	25.9	6 873	15.1	1 871	4.1	25 084	55.0
Female	143 525	39 611	27.6	19 367	13.5	5 313	3.7	79 234	55.2
**Birthplace**									
Sweden	156 324	41 119	26.3	22 310	14.3	5 998	3.8	86 897	55.6
Nordic not Sweden	4 062	1 010	24.9	455	11.2	210	5.2	2 387	58.8
EU28 not Nordic	5 139	1 416	27.6	610	11.9	183	3.6	2 930	57.0
Europe not EU28	5 375	1 511	28.1	783	14.6	208	3.9	2 873	53.5
Other birthplace	18 250	6 352	34.8	2 082	11.4	585	3.2	9 231	50.6
**Area of residence**									
Urban	145 212	42 871	29.5	19 185	13.2	5 271	3.6	77 885	53.6
Peri-urban	16 198	2 986	18.4	2 781	17.2	684	4.2	9 747	60.2
Rural	27 740	5 551	20.0	4 274	15.4	1 229	4.4	16 686	60.2
**Region of residence** ^**2**^									
Stockholm	46 578	24 401	52.4	2 133	4.6	1 335	2.9	18 709	40.2
Uppsala	7 193	1 705	23.7	1 358	18.9	238	3.3	3 892	54.1
Södermanland ^3^	4 704	117	-	991	-	257	-	3 339	-
Östergötland	8 000	1 174	14.7	1 555	19.4	346	4.3	4 925	61.6
Jönkoping	6 235	486	7.8	1 042	16.7	343	5.5	4 364	70.0
Kronoberg ^3^	3 435	20	-	642	-	88	-	2 685	-
Kalmar	4 360	925	21.2	664	15.2	182	4.2	2 589	59.4
Gotland	1 179	323	27.4	246	20.9	39	3.3	571	48.4
Blekinge	2 566	130	5.1	632	24.6	129	5.0	1 675	65.3
Skåne	24 271	1 435	5.9	5 015	20.7	813	3.3	17 008	70.1
Halland ^3^	5 574	82	-	1 087	-	363	-	4 042	-
Västra götaland	33 888	13 166	38.9	3 876	11.4	1 250	3.7	15 596	46.0
Värmland	4 810	1 933	40.2	724	15.1	183	3.8	1 970	41.0
Örebro	4 953	89	1.8	1 013	20.5	108	2.2	3 743	75.6
Västmanland	5 204	638	12.3	1 055	20.3	266	5.1	3 245	62.4
Dalarna	5 600	2 508	44.8	735	13.1	199	3.6	2 158	38.5
Gävleborg	4 682	59	1.3	926	19.8	272	5.8	3 425	73.2
Västernorrland	5 072	1 873	36.9	637	12.6	221	4.4	2 341	46.2
Jämtland	2 158	58	2.7	467	21.6	133	6.2	1 500	69.5
Västerbotten ^3^	4 510	33	-	787	-	247	-	3 443	-
Norrbotten	4 178	253	6.1	655	15.7	172	4.1	3 098	74.2

^1^ Primary healthcare data were only available for the years 2015 to 2017 for 17 of 21 regions

^2^ Regional values refer to the region of primary residence for study participants diagnosed with migraine, not the region of the health facility where the diagnosis occurred

^3^ Primary healthcare data were not available for these regions. The totals presented refer to the number of people with primary residence in the region receiving a migraine diagnosis at a primary health care visit that took place outside that region

Among people who were first diagnosed with migraine in a primary care visit, a higher proportion were in the youngest versus oldest age group (38.0% versus 18.3%), urban versus rural residents (29.5% versus 20.0%), living in the least versus most deprived areas (31.8% and 26.4%), and people born outside Europe versus native-born Swedes (34.8% versus 26.3%). In contrast, among people who received prescription drugs without a diagnosis recorded in 2015–2017, a higher proportion were older, lived in rural or peri-urban areas, and were born within the European Union (EU28).

There was also significant regional variation in the distribution of first migraine diagnoses across sources of care. The regions with the highest proportion of migraine cases first diagnosed in primary care included Stockholm (52.4%), Dalarna (44.8%), and Värmland (40.2%). Other regions with major urban centers showed large variations in the distribution by source of care (primary care, specialist outpatient, inpatient admission or prescription drug alone) including Stockholm (52.4%, 4.6%, 2.9%, 40.2%), Västra Götaland (38.9%, 11.4%, 3.7%, 46.0%), Skåne (5.9%, 20.7%, 3.3%, 70.1%) and Uppsala (23.7%, 18.9%, 3.3%, 54.1%).

## Discussion

### Principle findings

In this nationwide register-based study including 8,528,198 participants, we estimated a migraine rate of 43.7 cases per 1,000 persons ten years or older in Sweden in 2015 to 2023. There was higher migraine prevalence for people living in the least versus the most deprived areas although with a small effect estimate. Associations between migraine with sex and urban residence differed across small-area deprivation levels. We found significant variation in the source of first migraine diagnosis by region and personal characteristics.

### Comparisons with other studies

The migraine rate reported in this study is substantially lower than the previous estimate of at least one in eight (13%) people affected by migraine in Sweden [5]. The lower estimate is probably due to different data sources and population age groups used to measure prevalence across studies. The earlier estimate employed a population survey among people aged 18 to 74 years using a standardized questionnaire to ascertain migraine at a population level according to the International Headache Society (IHS) criteria. In this survey, it was further reported that half of respondents who fulfilled IHS migraine criteria had not been diagnosed by a physician [5].

In contrast, the current study relied on national and regional healthcare registers that captured only people who sought care and were diagnosed and/or treated for migraine. In addition, migraines are commonly managed at primary care level in Sweden and primary care data for this analysis was only available for 17 of 21 regions in the years 2015 to 2017. Furthermore, a large proportion of migraine cases were ascertained from prescription registers alone. We included only drugs that did not have multiple indications, and the registers do not include over- the-counter drugs used in migraine, such as NSAIDs. For these reasons, this study underestimates migraine prevalence in the general Swedish population given the well-recognized healthcare gaps for migraine patients and use of a restrictive migraine medication list to ascertain cases from prescription registers [6]. Nevertheless, the analysis of administrative registers could help identify healthcare gaps for migraine patients in Sweden including variations in care by region and socioeconomic context.

First, a substantial variation in the source of first migraine diagnosis across regions and by personal characteristics was shown in the sub-analysis of years with primary care data available. For example, in Region Stockholm, about half of migraine patients were diagnosed in primary care compared to lower proportions in Regions Västra Götaland, Uppsala and Skåne, respectively. This merits further investigation to understand if these results are due to differential data quality across regions and/or variations in regional healthcare for migraine patients. At the same time, across all areas, people diagnosed with migraine in primary care were more often in the youngest age group, urban residents, or living in least deprived areas. These groups may be more likely to have better access to and increased contacts with primary health care in their local areas [23, 24]. Prior research from Sweden has demonstrated disparities in the management of migraine cases across regions and socioeconomic groups, and our findings underscore that previous evidence [5, 13, 14, 23].

Second, regression results showed that people living in least deprived areas had higher migraine prevalence than those in the most deprived areas, although regression coefficients were relatively small. This seems to contrast with previous studies, which generally report higher migraine prevalence among people with lower individual-level socioeconomic status [13, 25, 26]. However, our findings, based on the analysis of healthcare registers, may again reflect the problem of migraine underdiagnosis especially among people with lower socioeconomic status [5, 6, 13, 25]. People in higher socioeconomic groups typically have better access to healthcare services, including specialized care for headache disorders [23, 24, 26, 27]. They are also more likely to seek and receive a diagnosis for their symptoms, which would increase the occurrence (or risk) of migraine in these better-resourced areas [13, 26, 27]. Findings from the sensitivity analysis that excluded primary healthcare data may further support this explanation since we did not identify a significant difference in migraine prevalence among people living in the least versus most deprived areas in contrast to the main analysis.

The results in this study showing stronger relative migraine likelihoods for women, older age groups, and urban residents in the most deprived versus least deprived areas could further underscore this explanation. For example, it could be that rural residents in the most deprived areas are even less connected to the healthcare system, making the relative migraine associations for urban residents stronger in these areas compared to those found in the least deprived areas. The same hypothesis could be considered for men versus women in areas with different deprivation levels and for the youngest age group (under 20 years) compared to older age groups across deprivation levels. These findings merit further investigation since they could point to socioeconomic inequalities in care seeking and management of migraine patients within Sweden as reported in previous research [13].

### Strengths and limitations of this study

This study leveraged multiple national and regional registers to enable research on a whole population basis providing greater statistical power and allowing for analysis by subpopulation groups. There are also some study limitations. First, migraine prevalence estimates presented in this paper underestimate the migraine burden in Sweden at a population level given well-recognized healthcare gaps for migraine patients resulting in undercounting and misclassification of cases. Age of migraine diagnosis is also typically higher than ten years old, which could further result in a lower-than-expected prevalence estimate. Second, regional primary care registers were available for only 17 of 21 regions (89% of the Swedish population) in the years 2015 to 2017, which may further contribute to underestimation of migraine prevalence in our cohort. In addition, a large proportion of cases were ascertained from prescription registers alone and migraine medications did not include drugs with multiple indications or that are sold without a prescription, such as analgesics. This could further undercount cases. Third, contextual factors such as small-area deprivation have measurement challenges including defining the area or developing indicators, and composite deprivation measures could obscure differences in areas even within the same deprivation level. Fourth, small-area deprivation level for each DeSO was measured in 2015 and the quartile grouping was assumed to remain unchanged throughout the study period. Finally, estimates were not adjusted for individual-level socioeconomic characteristics (e.g., income or education) although the small-area deprivation measure assigns the area-level value to each individual residing in the area. While this could increase risks of ecological fallacy, interpretations of results have focused on associations between migraine and area-level deprivation.

## Conclusions

This analysis of national and regional healthcare registers showed lower rates of diagnosed or treated migraine compared to population-level estimates of migraine prevalence underscoring potential healthcare gaps for migraine patients in Sweden. Higher prevalence of migraine among people in better-resourced areas using healthcare registry data may suggest better symptom recognition, care-seeking practices, and/or migraine management compared to those in less-resourced areas. Substantial variation in the source of first migraine diagnosis by region and personal characteristics merits further investigation. A more consistent approach to care seeking and management of migraine symptoms is needed in Sweden to reduce healthcare gaps and uneven practices by region and socioeconomic context.

## Supplementary Information

Below is the link to the electronic supplementary material.

Supplementary Material 1

## Data Availability

Due to ethical restrictions, the individual level data used in this study are not publicly available. However, the data supporting the findings of this study can be obtained from Statistics Sweden (www.scb.se) and the Swedish National Board of Health and Welfare (www.socialstyrelsen.se)..
