# Supplementary material for: Geographical patterns and determinants of migraine in persons aged ten years or older living in Sweden from 2015 to 2023: a nationwide cross-sectional study
Source: J Headache Pain. 2026 Feb 19;27(1):50. doi: 10.1186/s10194-026-02287-1 (PMC12918554; doi:10.1186/s10194-026-02287-1)
Supplement: Supplementary file 1 — Supplementary Material 1 [file 10194_2026_2287_MOESM1_ESM.docx]

| **Title** | **Geographical patterns and determinants of migraine in persons aged ten years or older living in Sweden from 2015 to 2023: a nationwide cross-sectional study**  **SUPPLEMENTARY MATERIALS** |
| --- | --- |
| **Author names** | Emily White Johansson^1, 2 *^  Ahmed Nabil Shaaban^1*^  Mattias Linde^3, 4^  Mathias Mattson^1^  Lode van der Velde^1^  Sofie Gustafsson^1, 5^  Johan Holm^5^  Christina Dalman^1^  Emilie E Agardh^1^  * Both authors contributed equally |
| **Author information** | 1. Department of Global Public Health, Karolinska Institutet, Stockholm, Sweden 2. Global Health and Migration Unit, Department of Women’s and Children’s Health, Uppsala University, Uppsala, Sweden 3. Department of Neuromedicine and Movement Science, Norwegian University of Science and Technology (NTNU), Trondheim, Norway 4. Regional Migraine Unit, Sahlgrenska University Hospital, Gothenburg, Sweden 5. Pfizer AB, Stockholm, Sweden |
| **Corresponding author** | Emily White Johansson PhD  Associate Professor  Uppsala University  Department of Women’s and Children’s Health  Global Health and Migration Unit  Akademiska sjukhuset  751 85 Uppsala, Sweden  Email: [emily.johansson@uu.se](mailto:emily.johansson@uu.se)  ORCID ID: 0000-0001-5162-8277  Karolinska Institutet  Department of Global Public Health |

**Supplementary materials**

Table S1: ICD-10-SE and ATC codes for migraine measurement

Table S2: P-values for interaction terms between small-area deprivation and other covariates

Table S3: Diagnosed migraine rates per 1,000 persons aged ten years or older in Sweden in 2015-2023 *(excludes migraine ascertained from prescribed treatment alone)*

Table S4: Characteristics of study participants with or without diagnosed migraine *(excludes migraine ascertained from prescribed treatment alone)*

Table S5: Association between small-area deprivation and other covariates on diagnosed migraine among study participants *(excludes migraine ascertained from prescribed treatment alone)*

Table S6: Association between small-area deprivation and other covariates on migraine among study participants *(excludes migraine ascertained from primary healthcare data)*

Table S1: ICD-10-SE and ATC codes for migraine measurement

| **Migraine exposure measurement** | | |
| --- | --- | --- |
| **Diagnosis (ICD-10-SE) codes** | | |
| Migraine | G43.0 | Migraine without aura |
| Migraine | G43.1 | Migraine with aura |
| Migraine | G43.2 | Status migrainosus |
| Migraine | G43.3 | Complicated migraine |
| Migraine | G43.8 | Other specified migraines |
| Migraine | G43.9 | Migraine, unspecified |
| **Prescription (ATC) codes** | | |
| CGRP | N02CD01 | Erenumab |
| CGRP | N02CD02 | Galkanezumab |
| CGRP | N02CD03 | Fremanezumab |
| CGRP | N02CD05 | Eptinezumab |
| CGRP | N02CD06 | Rimegepant |
| GGRP | N02CD07 | Atogepant |
| Triptan | N02CC01 | Sumatriptan |
| Triptan | N02CC02 | Naratriptan |
| Triptan | N02CC03 | Zolmitriptan |
| Triptan | N02CC04 | Rizatriptan |
| Triptan | N02CC05 | Almotriptan |
| Triptan | N02CC06 | Eletriptan |

Table S2: P-values for the interaction terms between small-area deprivation level and other covariates

| **Small-area deprivation and area of residence** | | |
| --- | --- | --- |
| Very low deprivation | Urban | <0.001 |
| Very low deprivation | Peri-urban | <0.001 |
| Low deprivation | Urban | <0.001 |
| Low deprivation | Peri-urban | <0.001 |
| High deprivation | Urban | 0.316 |
| High deprivation | Peri-urban | 0.063 |
| **Small-area deprivation and sex** | | |
| Very low deprivation | Female | <0.001 |
| Low deprivation | Female | <0.001 |
| High deprivation | Female | 0.011 |
| **Small-area deprivation and birthplace** | | |
| Very low deprivation | Nordic outside Sweden | <0.001 |
| Very low deprivation | EU28 outside Nordic countries | 0.007 |
| Very low deprivation | Europe outside EU28 | 0.508 |
| Very low deprivation | Other birthplace | 0.001 |
| Low deprivation | Nordic outside Sweden | 0.822 |
| Low deprivation | EU28 outside Nordic countries | 0.144 |
| Low deprivation | Europe outside EU28 | 0.832 |
| Low deprivation | Other birthplace | 0.002 |
| High deprivation | Nordic outside Sweden | 0.223 |
| High deprivation | EU28 outside Nordic countries | 0.287 |
| High deprivation | Europe outside EU28 | 0.666 |
| High deprivation | Other birthplace | 0.112 |
| **Small-area deprivation and age** | | |
| Very low deprivation | Age (continuous) | <0.001 |
| Low deprivation | Age (continuous) | 0.003 |
| High deprivation | Age (continuous) | <0.001 |

Table S3: Diagnosed migraine rates per 1,000 persons aged ten years or older in Sweden in 2015-2023 *(excludes migraine ascertained from prescribed treatment alone)*

|  | Age-standardized rate | Crude rate | Migraine cases | Persons aged 10 years or older on 31 December 2023 |
| --- | --- | --- | --- | --- |
|  | per 1 000 | per 1 000 | N | N |
| **National** | **18.2** | **18.2** | **155 216** | **8 528 198** |
| **Small-area deprivation** |  |  |  |  |
| Very low deprivation | 19.5 | 19.7 | 44 282 | 2 251 388 |
| Low deprivation | 18.8 | 18.7 | 40 893 | 2 192 330 |
| High deprivation | 17.5 | 17.1 | 36 064 | 2 113 810 |
| Very high deprivation | 17.0 | 17.2 | 33 977 | 1 970 670 |
| **Area of residence** |  |  |  |  |
| Urban | 18.6 | 18.9 | 121 784 | 6 427 223 |
| Peri-urban | 17.2 | 16.7 | 12 314 | 737 910 |
| Rural | 16.8 | 15.5 | 21 118 | 1 363 065 |
| **Region of residence** |  |  |  |  |
| Stockholm | 23.8 | 24.7 | 48 020 | 1 945 231 |
| Uppsala | 19.2 | 19.5 | 6 086 | 312 429 |
| Södermanland ^1^ | 15.3 | 14.8 | 3 615 | 244 114 |
| Östergötland | 16.4 | 16.4 | 6 349 | 388 142 |
| Jönkoping | 14.7 | 14.5 | 4 367 | 300 653 |
| Kronoberg ^1^ | 13.2 | 13.1 | 2 131 | 163 226 |
| Kalmar | 16.3 | 15.3 | 3 111 | 203 473 |
| Gotland | 23.2 | 21.4 | 1 084 | 50 608 |
| Blekinge | 15.9 | 15.4 | 2 040 | 132 535 |
| Skåne | 14.2 | 14.3 | 15 942 | 1 117 910 |
| Halland ^1^ | 14.8 | 14.5 | 4 039 | 278 930 |
| Västra götaland | 19.1 | 19.2 | 27 481 | 1 431 275 |
| Värmland | 19.9 | 18.9 | 4 453 | 235 738 |
| Örebro | 12.2 | 12.1 | 3 055 | 252 410 |
| Västmanland | 17.7 | 17.4 | 3 965 | 228 450 |
| Dalarna | 21.6 | 20.4 | 4 945 | 242 109 |
| Gävleborg | 13.3 | 12.8 | 3 090 | 241 845 |
| Västernorrland | 21.8 | 20.8 | 4 326 | 208 111 |
| Jämtland | 14.1 | 13.6 | 1 496 | 109 875 |
| Västerbotten ^1^ | 13.0 | 12.9 | 2 956 | 228 341 |
| Norrbotten | 13.1 | 12.5 | 2 665 | 212 793 |

**^1^** Primary healthcare data was not available for these regions. For these regions, crude and age-standardized migraine rates are based on inpatient and specialist outpatient diagnoses and prescribed drugs only. All other regions had primary healthcare data available for the years 2015 to 2017 only.

Table S4: Characteristics of study participants with or without diagnosed migraine *(excludes migraine ascertained from prescribed treatment alone)*

|  | **Study participants** | | | |
| --- | --- | --- | --- | --- |
|  | **Migraine** | | **No Migraine** | |
|  | **N** | **%** | **N** | **%** |
| **Total** | **155 216** | **1.8** | **8 372 982** | **98.2** |
| **Small-area deprivation level** |  |  |  |  |
| Very low deprivation | 44 282 | 2.0 | 2 207 106 | 98.0 |
| Low deprivation | 40 893 | 1.9 | 2 151 437 | 98.1 |
| High deprivation | 36 064 | 1.7 | 2 077 746 | 98.3 |
| Very high deprivation | 33 977 | 1.7 | 1 936 693 | 98.3 |
| **Area type of residence** |  |  |  |  |
| Urban | 121 784 | 1.9 | 6 305 439 | 98.1 |
| Peri-urban | 12 314 | 1.7 | 725 596 | 98.3 |
| Rural | 21 118 | 1.6 | 1 341 947 | 98.5 |
| **Age, median (IQR)** | 45 | (32-58) | 48 | (30-65) |
| **Sex** |  |  |  |  |
| Male | 39 190 | 0.9 | 4 211 561 | 99.1 |
| Female | 116 026 | 2.7 | 4 161 421 | 97.3 |
| **Birthplace** |  |  |  |  |
| Sweden | 128 981 | 1.8 | 7 055 718 | 98.2 |
| Nordic not Sweden | 2 929 | 1.6 | 178 695 | 98.4 |
| EU28 not Nordic | 4 109 | 1.7 | 245 607 | 98.4 |
| Europe not EU28 | 4 283 | 2.1 | 203 771 | 97.9 |
| Other birthplace | 14 914 | 2.1 | 689 191 | 97.9 |

Table S5: Association between small-area deprivation and other covariates on diagnosed migraine among study participants *(excludes migraine ascertained from prescribed treatment alone)*

|  | **Study participants** | | | | | | | |
| --- | --- | --- | --- | --- | --- | --- | --- | --- |
|  | **Crude** | | | | **Adjusted** | | | |
|  | OR | 95% CI | | pvalue | OR | 95% CI | | pvalue |
| **Small-area deprivation** |  |  |  |  |  |  |  |  |
| Very low deprivation | 1.14 | 1.13 | 1.16 | <0.001 | 1.18 | 1.16 | 1.19 | <0.001 |
| Low deprivation | 1.08 | 1.07 | 1.10 | <0.001 | 1.15 | 1.13 | 1.17 | <0.001 |
| High deprivation | 0.99 | 0.97 | 1.00 | 0.161 | 1.06 | 1.04 | 1.07 | <0.001 |
| Very high deprivation | 1.00 |  |  |  | 1.00 |  |  |  |
| **Area type of residence** |  |  |  |  |  |  |  |  |
| Urban | 1.23 | 1.21 | 1.25 | <0,001 | 1.15 | 1.13 | 1.17 | <0.001 |
| Peri-urban | 1.08 | 1.05 | 1.10 | <0.001 | 1.04 | 1.02 | 1.07 | <0.001 |
| Rural | 1.00 |  |  |  | 1.00 |  |  |  |
| **Age (continuous)** | 0.99 | 0.99 | 0.99 | <0.001 | 0.99 | 0.99 | 0.99 | <0.001 |
| **Sex** |  |  |  |  |  |  |  |  |
| Male | 1.00 |  |  |  | 1.00 |  |  |  |
| Female | 3.00 | 2.96 | 3.03 | <0.001 | 3.02 | 2.99 | 3.06 | <0.001 |
| **Birthplace** |  |  |  |  |  |  |  |  |
| Sweden | 1.00 |  |  |  | 1.00 |  |  |  |
| Nordic not Sweden | 0.90 | 0.86 | 0.93 | <0.001 | 0.94 | 0.90 | 0.97 | 0.001 |
| EU28 not Nordic | 0.92 | 0.89 | 0.94 | <0.001 | 0.95 | 0.92 | 0.98 | 0.001 |
| Europe not EU28 | 1.15 | 1.11 | 1.19 | <0.001 | 1.19 | 1.15 | 1.23 | <0.001 |
| Other birthplace | 1.18 | 1.16 | 1.20 | <0.001 | 1.19 | 1.17 | 1.21 | <0.001 |

Table S6: Association between small-area deprivation and other covariates on migraine among study participants *(excludes migraine ascertained from primary healthcare data)*

|  | **Crude** | | | | **Adjusted** | | | |
| --- | --- | --- | --- | --- | --- | --- | --- | --- |
|  | OR | 95% CI | | pvalue | OR | 95% CI | | pvalue |
| **Small-area deprivation** |  |  |  |  |  |  |  |  |
| Very low deprivation | 0.97 | 0.96 | 0.98 | <0.001 | 1.00 | 0.99 | 1.01 | 0.684 |
| Low deprivation | 1.01 | 1.00 | 1.02 | 0.131 | 1.05 | 1.04 | 1.06 | <0.001 |
| High deprivation | 1.01 | 1.00 | 1.02 | 0.139 | 1.05 | 1.04 | 1.06 | <0.001 |
| Very high deprivation | 1.00 |  |  |  | 1.00 |  |  |  |
| **Area of residence** |  |  |  |  |  |  |  |  |
| Urban | 1.07 | 1.06 | 1.08 | <0.001 | 1.03 | 1.01 | 1.04 | <0.001 |
| Peri-urban | 1.10 | 1.08 | 1.11 | <0.001 | 1.08 | 1.06 | 1.10 | <0.001 |
| Rural | 1.00 |  |  |  | 1.00 |  |  |  |
| **Age (continuous)** | 1.00 | 1.00 | 1.00 | <0.001 | 1.00 | 1.00 | 1.00 | <0.001 |
| **Sex** |  |  |  |  |  |  |  |  |
| Male | 1.00 |  |  |  | 1.00 |  |  |  |
| Female | 3.06 | 3.03 | 3.08 | <0.001 | 3.08 | 3.05 | 3.10 | <0.001 |
| **Birthplace** |  |  |  |  |  |  |  |  |
| Sweden | 1.00 |  |  |  | 1,00 |  |  |  |
| Nordic not Sweden | 0.87 | 0.85 | 0.90 | <0.001 | 0.87 | 0.84 | 0.89 | <0.001 |
| EU28 not Nordic | 0.97 | 0.95 | 0.99 | 0,002 | 0.98 | 0.96 | 1.00 | 0.119 |
| Europe not EU28 | 1.22 | 1.19 | 1.24 | <0.001 | 1.23 | 1.20 | 1.26 | <0.001 |
| Other birthplace | 1.21 | 1.19 | 1.22 | <0.001 | 1.21 | 1.19 | 1.22 | <0.001 |
